# 48352 Mechanisms Underlying Lipidomic Changes in Major Depressive Disorder

**DOI:** 10.1017/cts.2021.656

**Published:** 2021-03-30

**Authors:** Ana Paula Costa, Milenna T. van Dijr, Ardesheer Talati, Myrna M. Weissmann, Laura Beth McIntire

**Affiliations:** Columbia University

## Abstract

ABSTRACT IMPACT: Lipidomics is emerging as a powerful strategy to identify biomarkers for Major Depressive Disorder, as well as therapeutic targets in lipid metabolic pathways. OBJECTIVES/GOALS: Lipidomics is increasingly recognized in precision psychiatry for global lipid perturbations in patients suffering from Major Depressive Disorder (MDD). We will test the hypothesis that lipid metabolism dysregulation is associated with familial risk of depression. METHODS/STUDY POPULATION: Patients with MDD (G1), children (G2), and grandchildren (G3) have been part of a longitudinal study since 1982. If a parent G2 and grandparent G1 have MDD, G3 is considered a high risk of depression. Biospecimens (saliva and serum) were collected for full exome sequencing and RNA analysis. Samples will also be extracted for lipid content and lipids will be identified by mass spectrometry. A panel of nearly 600 lipid species can reliably be identified and quantified using liquid chromatography paired with tandem mass spectrometry (LC-MS/MS). Dysregulated lipids will be correlated with familial risk of depression in samples of G3. RESULTS/ANTICIPATED RESULTS: We hypothesize that dysregulation of lipids and lipid metabolism will be apparent in biospecimens from the high risk compared to the low risk of depression. Also, alterations in RNA transcriptomics of genes involved in lipid metabolic networks are associated with familial risk of depression. Several differential lipid species were previously identified to be associated with MDD. Reduced phosphatidylcholine(PC), phosphatidylethanolamine(PE), phosphatidylinositol(PI), and increased LysoPC, LysoPE, ceramide, triacylglycerol, and diacylglycerol levels have been correlated to MDD. However, these results need to be replicated in independent studies using lipidomics analysis. DISCUSSION/SIGNIFICANCE OF FINDINGS: It is highly likely that completely novel cellular targets will emerge from these studies by uncovering the convergence of lipidomics and genetic variance of lipid metabolic enzymes as biomarkers for predisposition to MDD as well as potential targets for therapeutic development for MDD.

